# Spatial and Temporal Dynamics of Bentho-Demersal Communities From Bottom Trawl Across the Moroccan Mediterranean Coast in Relation to Environmental Conditions: Implications for Fisheries Management

**DOI:** 10.1155/sci5/5574051

**Published:** 2025-02-24

**Authors:** Douaa Slimani, Souad Abdellaoui, Najib El Ouamari, Khaoula Kasmi, Rajae Mouedden, Imade Ouebdil, Nassir Kaddouri, Jamal Settih, Mostafa Layachi, Mohamed Fadili, Khalid Chaabane

**Affiliations:** ^1^Department of Biology, Laboratory for the Improvement of Agricultural Production, Biotechnology and the Environment, FSO, Mohammed First University, Oujda, Morocco; ^2^Fishing Laboratory, Regional Center of the National Institute of Fisheries Research, Casablanca, Morocco; ^3^Fishing Laboratory, Regional Center of the National Institute of Fisheries Research, Nador, Morocco; ^4^Department of Geography, Dynamic Laboratory of Arid Environments, Planning and Regional Development, FLSH, Mohammed First University, Oujda, Morocco; ^5^Biodiversity, Ecology and Genomics Laboratory, Department of Biology, Faculty of Sciences, Mohammed V University, Rabat, Morocco

**Keywords:** community structure, environmental factors, fisheries management, Moroccan Mediterranean, species diversity

## Abstract

Understanding the temporal and spatial patterns, as well as habitat preferences, of marine communities is crucial for interpreting ecosystem functioning and effectively protecting marine organisms' resources. In this paper, we investigated the changes in marine communities in the Moroccan Mediterranean (20–620 m depth) over both space and time. Using data from trawl scientific surveys conducted by the National Fisheries Research Institute from 2018 to 2021, we conducted a quantitative analysis to (1) assess the seasonal spatial patterns of bentho-demersal communities, including both economically important species and those not directly targeted by fisheries, and (2) explore the interactions between these communities and environmental factors to gain deeper insights into the dynamics of communities structure. We selected several environmental characterizations including depth, sea surface temperature, bottom temperature, salinity, chlorophyll-a, dissolved oxygen, pH, and bottom seabed nature. Depth was the predominant factor responsible for most of the variation observed in both ecological parameters and the composition of bentho-demersal species. The study largely confirms that bentho-demersal communities along the Moroccan Mediterranean coast respond to their environment, displaying a structural pattern shaped by an offshore to inshore environmental gradient, and exhibiting low seasonal variations. These results have significant implications for fisheries management, offering crucial insights into the parameters influencing the distribution of bentho-demersal communities. This is particularly relevant for mixed-species fisheries, just like those operating in the Moroccan Mediterranean trawl fishery.

## 1. Introduction

Marine ecosystems rely heavily on biodiversity, which plays a key role in shaping their structure and functioning. This biodiversity also provides valuable services and benefits that contribute to the health, social well-being, and prosperity of human societies on local, regional, and global scales [1, 2]. Globally, organisms are disappearing at an alarming rate, driven by climate change and human impacts, which are causing an ongoing decline in biodiversity [3]. These trends have generated widespread concerns and considerable interest in the effects of biodiversity loss on ecosystems. Preserving marine biodiversity [4] necessitates a comprehensive understanding of biogeographic patterns and temporal variability of marine species and communities that define the limits of a species' distribution, as it can offer valuable insights into the biodiversity–ecosystem functioning relationships to support scientific ecological conservation and management [5]. A fundamental prerequisite for creating effective spatial management models pertinent to biological and ecological processes involves comprehending species–environment relationships. These relationships are essential tools for understanding and predicting species richness and abundance across different environments [6]. By examining how species or communities respond to habitat components, one can pinpoint the specific attributes that establish the physical and ecological boundaries governing their distribution.

The Mediterranean Sea (located between 30°N–46°N latitude and 6°W–36°E longitude) stands out as a critical hotspot for marine biodiversity [7] and is recognized as a severely impacted marine ecosystems globally [8]. In the current era of global change, most Mediterranean marine biodiversity is undergoing unprecedented alterations at multiple levels due to the effects of unsustainable and environmentally damaging human activities, including overexploitation, ecosystem destruction, and pollution, impacting the temporal dynamics of communities and ecosystem functions [7]. Uncontrolled climate change and ocean acidification pose additional threats, now recognized as primary drivers of marine biodiversity loss. Their impact goes beyond the simple abundance of target species, shaping species assemblages, altering the dynamics of food webs, and ultimately impacting the productivity and stability of marine ecosystems [9], suggesting that we are in a biodiversity crisis. According to Food and Agriculture Organization [10] and the General Fisheries Commission for the Mediterranean [11], most of stocks targeted for commercial exploitation in the Mediterranean Sea are currently heavily fished or overexploited. Additionally, a number of noncommercial benthic and demersal species have seen reductions in both their population numbers and biomass, along with a decrease in biodiversity [12]. Specifically, advancements in fishing technologies and intensified fishing efforts to meet the increasing seafood demand of a growing global population are exerting significant pressure on the marine resources within the Mediterranean. Thus, fishing activities, particularly bottom trawling, are considered as significant factors potentially impacting entire habitats and the ecosystem, influencing the structure and composition of the seabed and their associated benthic and demersal communities [13, 14] and so, monitoring the community composition and spatio-temporal distribution of marine ecosystems over time is crucial for assessing potential changes in their structure. Hence, research should not only focus on changes in species composition but also on changes in community function and the relationships between functional diversity and distributions over time. It is vital to comprehend the fundamental causes of diversity patterns and how they interact with environmental factors to effectively conserve and manage ecosystems. This understanding ensures that conservation strategies are informed and aimed at maintaining ecological stability and health [15]. For this reason, the diversity of ecological communities, along with the spatial distribution of marine species, has been extensively studied across different ecosystems worldwide. These studies have been conducted in various contexts, using both annual and aggregated temporal resolutions to capture a more comprehensive understanding of species dynamics and environmental interactions over time. According to available findings, the variations in abundance and/or geographic distribution of species can be attributed to not only the impact of biotic factors like distribution and abundance of preys and predators [16–18], but also favorable environmental factors like temperature, salinity, sediment composition, appropriate depth ranges, and topography [19–30]. For instance, temperature and depth have been identified as crucial factors shaping the distribution and interactions of marine species [31–37]. Furthermore, other research revealed a strong and positive correlation between sea surface chlorophyll-a concentration and the catches and distribution of certain species [38].

Given the dynamic nature of the Mediterranean Sea, which showcases clear variations in response to diverse environmental spatial factors, and likely have impacts on the dynamics of the whole community, including the bentho-demersal community [39], the interplay of these spatial factors can influence species distribution, interactions, and the overall structure of the ecosystem, highlighting the importance of understanding such environmental influences in this region. However, acquiring detailed spatial information remains challenging in many regions. Studies exploring the temporal and spatial variations on the functional structure and distribution of marine communities in the western Mediterranean Sea are relatively limited. Biological research on commercial fisheries has historically centered on aspects such as behavior, age and growth, and reproduction. However, it is only recently that distribution and abundance data has become a significant focus in the literature.

Analyzing the spatial distribution patterns of the bentho-demersal community in the western Mediterranean Sea was the primary goal of this study, focusing on three major groups: fishes, cephalopods, and crustaceans. By examining these patterns of a heavily exploited region, we sought to gain insights into how the community structure varies spatially and temporally. To achieve this, we investigated species abundance, biomass, and biodiversity along the coast of Morocco. Our goal was to identify the factors that most strongly predict spatial patterns, in order to deepen our understanding of how environmental conditions influence the dynamics of bentho-demersal communities in this region.

## 2. Materials and Methods

### 2.1. Bentho-Demersal Community Sampling

**Figure 1:**
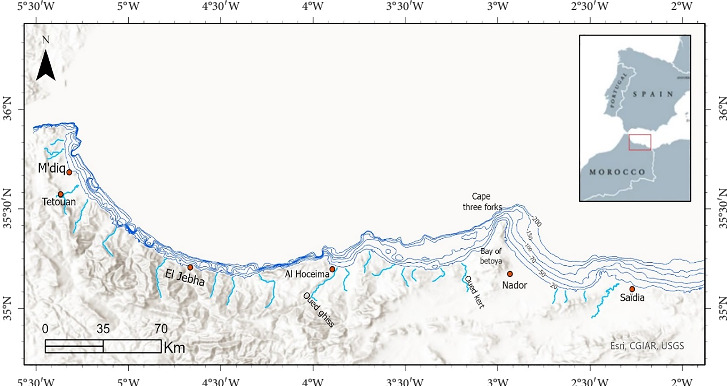
Studying area, Moroccan Mediterranean.

Eight scientific trawling surveys conducted by the National Fisheries Research Institute (INRH) provided the biological data for this study. During these surveys, samples were collected seasonally from 60 fixed sampling sites per survey aboard the oceanographic vessel “CHARIF AL IDRISSI” from 2018 to 2021 (Figure 1). The sampling sites were randomly located across the region, covering depths from 20 to 620 m, predominantly in areas with substantial fishing activity. Each trawl operation lasted between 30 and 60 min. During the surveys, each haul was thoroughly processed to classify and identify all marine organisms collected. In addition to the biological data, a comprehensive set of data was also collected, including detailed records related to the characteristics of each haul, the specific fishery units involved, and the geographic coordinates of the fishing locations.

### 2.2. Numerical Data Analysis and Statistical Methods

We used survey data to calculate ecological metrics for the bentho-demersal community, including all species of fishes, cephalopods, and crustaceans. These metrics include abundance (the number of individuals collected per hour), biomass (the total weight caught per hour measured in kilograms), species richness (number of species encountered per haul), and the Shannon index (H′; a quantitative measure of biodiversity). Species richness was selected for this study as it offers a clear and uncomplicated way to quantify the diversity within a community. In addition to species richness, the Shannon index was employed.

To analyze the data, we initially computed species abundances, biomasses, and biodiversity indices for each sampling station, then, we conducted these calculations separately for each season and for each species group (fishes, cephalopods, and crustaceans). Overall, we computed the mean values for abundance, biomass, and biodiversity, along with their respective standard deviations (SDs) and medians, to offer a thorough overview of the biological data for each species group.

To evaluate for any significance of differences in abundance, biomass, and biodiversity between the two seasons, we employed Kruskal–Wallis statistical tests. This nonparametric test was conducted for the entire bentho-demersal community, allowing for a broad understanding of the ecosystem's structure and dynamics. In addition, separate analyses were performed for distinct groups within the community, including fishes, cephalopods, and crustaceans, to provide detailed insights into each group's specific characteristics and interactions. Furthermore, in cases where the assumption of homogeneity of variance required for the Kruskal–Wallis test was not met, we utilize Welch tests to examine significant differences between the two seasons.

To emphasize the spatial distribution of the community within the study area, the ecological information from each sampled station, in both seasons and by group, were integrated into Arc Geographic Information System (GIS), enabled us to generate comprehensive cartographic representations.

We also investigated how the distribution of the bentho-demersal assemblage, considered both as a whole and separately for fishes, cephalopods, and crustaceans, responds to various environmental factors. To do this, we employed nine explanatory variables including (i) SST (°C), (ii) SBT (°C), (iii) sea surface salinity (SSS) (PPS), (iv) pH, (v) dissolved oxygen (DO) (mg/L), (vi) fluorescence (μg Chl a^−1^), (vii) seabed nature, (viii) depth (m), and (ix) season, to analyze their impact on abundance, biomass, and biodiversity (Table 1). Depth and seabed characteristics were recorded during the experimental surveys conducted each season. The remaining environmental variables were sourced from satellite data (https://data.marine.copernicus.eu/products).

### 2.3. Data Analysis

Spearman's correlation was used to evaluate relationships between environmental parameters as well as to verify for possible correlations between the measured abiotic parameters and the composition of the bentho-demersal assemblages. The similarity among the community structures was tested using the RELATE routine in PRIMER 6, based on the corresponding similarity matrices.

To evaluate the influence of environmental variables on the variability of bentho-demersal data (for the total community and for fishes, cephalopods, and crustaceans separated) and to select the best explanatory model, we applied a DistLM (Distance-Based Linear Model). DistLM is designed to determine how much of the variability is attributable to predictor indicators [40]. Environmental data were log-transformed (log (*x* + 1)) and biotic data were square root–transformed. Model selection was performed by examining all possible interactions of predictor variables, and based on three different measures: The best overall model was found using the Akaike Information Criterion (AIC) and Bayesian Information Criterion (BIC) in order to reveal the paramount combination of significant variables influencing the bentho-demersal community composition [40, 41]. Specifically, AIC and BIC were used as a measure for goodness of fit: the smaller the AIC and BIC values, the better the compromise among fit, parsimony, and predictive quality, containing only relevant predictors; i.e. those predictors with 95% credibility intervals not including zero. Both backward and forward stepwise approaches were adopted to test the importance of the independent variables.

A distance-based redundancy analysis (dbRDA) was associated to examine and illustrate graphically the relationship between predictor variables and the bentho-demersal community structure [40], based on triangular matrices of Bray–Curtis similarity of log (*x* + 1) transformed ecological data and standardized environmental data. In all analyses, 9999 permutations were made. The similarity index used for abundance data was the Bray Curtis coefficient. The PERMANOVA for environmental variables was run on the basis of the similarity matrix obtained by Euclidean distance. All these statistical calculations were done using PRIMER in PERMANOVA V.6 statistical software package [42, 43].

## 3. Results

As detailed in Table 2, the Kruskal–Wallis tests revealed significant seasonal variations in the abundance and biomass of the total community (*p* value < 0.05). However, this was not observed for the biomass of cephalopods and crustaceans, or the abundance of fish and crustaceans. No significant seasonal variations were observed in species richness or Shannon's index, with *p* values ≠ 0.05, except for the Shannon index for cephalopods (*p* value < 0.05).

In contrast, Welch tests identified seasonal variations in abundance and biomass for the total community, as well as for fish, cephalopods, and crustaceans, all with *p* values < 0.05. The Welch tests did not find significant differences in the Shannon index between seasons for any of the four groups studied.

**Figure 2:**
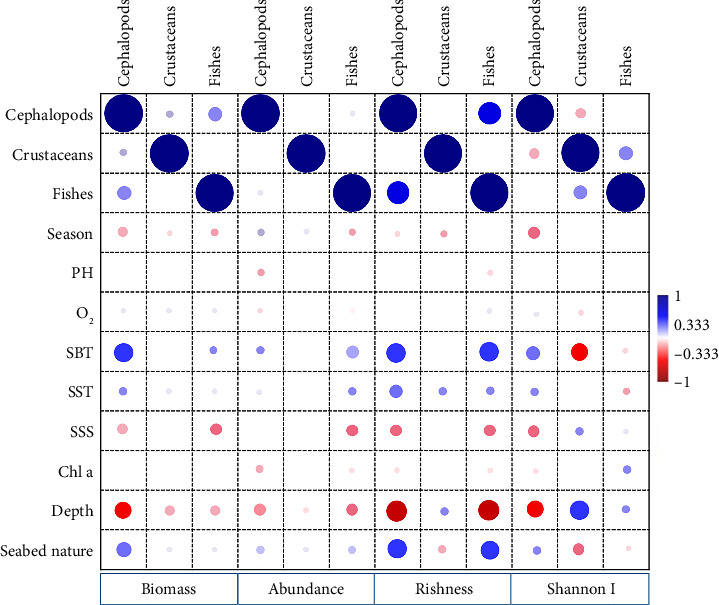
A correlation matrix computed separately for each group examining the relationships between environmental variables and ecological indexes along the Moroccan Mediterranean coast.

Depth, SST, SBT, SSS, and chlorophyll a were identified as significant predictors for the total community's abundance, biomass, and biodiversity indices (Table 3). Depth showed a notable negative correlation with abundance, biomass, and biodiversity indices, except for the Shannon index for crustaceans. This indicates that areas with greater depths generally had lower values for these biological metrics (Figure 2).

Models of fish abundance, biomass, and diversity identified temperature and salinity as significant predictors, although the Shannon index uniquely highlighted fluorescence as a key factor instead of temperature (Table 3). Both SST and SBT were positively correlated with fish abundance, biomass, and the richness index. In contrast, areas with higher biomass, abundance, and biodiversity of fish were associated with lower salinity levels, as indicated by the negative correlation observed with SSS (Figure 2).

For the cephalopod's community, depth and SBT emerged as significant predictors for all dependent variables (Table 3). Spearman's correlation analysis revealed positive relationship between both SST and SBT with cephalopod abundance, biomass, and biodiversity (Figure 2).

In the crustacean models, depth and temperature were identified as key predictors, similar to the cephalopod community (Table 3). Furthermore, depth was positively correlated with both species' richness and the Shannon index (Figure 2).

### 3.1. Estimated Distribution of Ecological Metrics of the Total Bentho-Demersal Community

**Figure 3:**
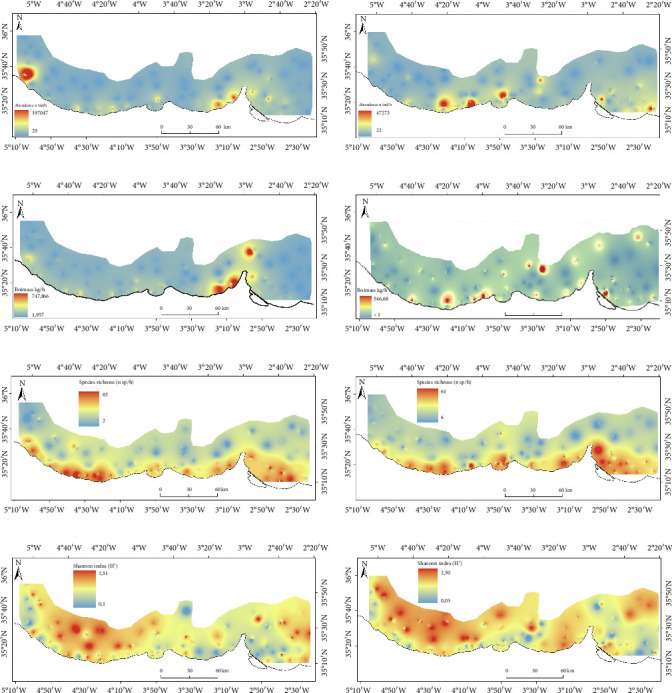
Total bentho-demersal community distribution of abundance ((a) winter and (b) summer), biomass ((c) winter and (d) summer), species richness ((e) winter and (f) summer), and Shannon index ((g) winter and (h) summer), respectively (refer to Figure 1 for further details on the study area).

In the western part of the study area, abundance was higher during winter (Figure 3(a)). Conversely, in summer, increased abundance was concentrated in the central and eastern regions, primarily on the continental shelf (Figure 3(b)). The distribution patterns for biomass were similar to those for abundance (Figures 3(c) and 3(d)). Higher values of the richness index were more widely dispersed near the continental shelf, with no significant seasonal differences observed in its distribution (Figures 3(e) and 3(f)). The Shannon index showed similar distribution patterns across seasons, with higher values found predominantly around M'diq and Al Hoceima (Figures 3(g) and 3(h)).

### 3.2. Estimated Distribution of Ecological Metrics of Fish Community

**Figure 4:**
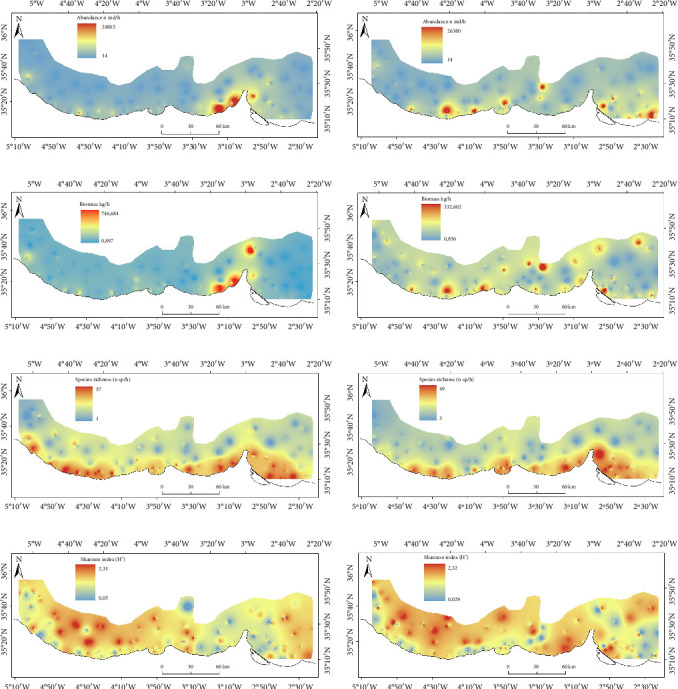
Fish community distribution of abundance ((a) winter and (b) summer), biomass ((c) winter and (d) summer), species richness ((e) winter and (f) summer), and Shannon index ((g) winter and (h) summer), respectively (refer to Figure 1 for further details on the study area).

Winter estimates of fish abundance were higher in the eastern part of the study area (Figure 4(a)). In summer, elevated abundance values were concentrated mainly in the central and eastern regions, particularly on the continental shelf (Figure 4(b)). The biomass distribution reflected the abundance patterns observed during summer (Figures 4(c) and 4(d)). Species richness estimates demonstrated an expansion from the continental shelf to the upper slope across the study area in both seasons (Figures 4(e)and 4(f)). The Shannon index distributions were consistent between winter and summer, with higher values found in the western part of the study area near the upper slope and in the central region (Figures 4(g) and 4(h)).

### 3.3. Estimated Distribution of Ecological Metrics of Cephalopods' Community

**Figure 5:**
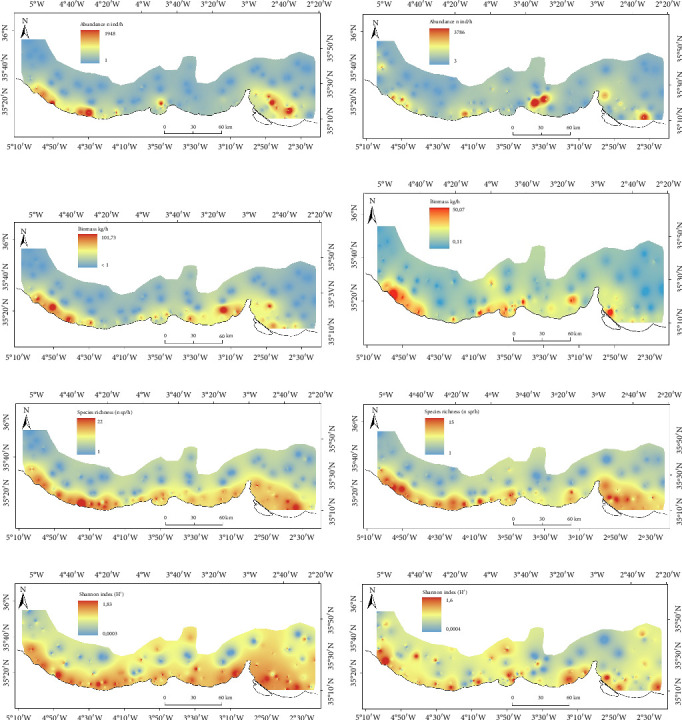
Cephalopods community distribution of abundance ((a) winter and (b) summer), biomass ((c) winter and (d) summer), species richness ((e) winter and (f) summer), and Shannon index ((g) winter and (h) summer), respectively (refer to figure 1 for further details on the study area).

During winter, higher abundance values were observed in coastal areas, particularly in the eastern and western regions (Figure 5(a)). In contrast, during summer, the central region displayed the highest abundance values (Figure 5(b)). The biomass distribution of cephalopods exhibited similar patterns across seasons, with elevated values found in the central and western parts during winter, and also in the eastern part during summer (Figures 5(c) and 5(d)). Richness estimates for cephalopods showed consistent patterns in both seasons, with higher values in coastal regions of the western and eastern areas (Figures 5(e) and 5(f)). The Shannon index for cephalopods also displayed similar seasonal distributions, although in winter, higher values extended from the continental shelf to the upper slope throughout the study area, while in summer, they were more concentrated near the shoreline (Figures 5(g) and 5(h)).

### 3.4. Estimated Distribution of Ecological Metrics of Crustaceans' Community

**Figure 6:**
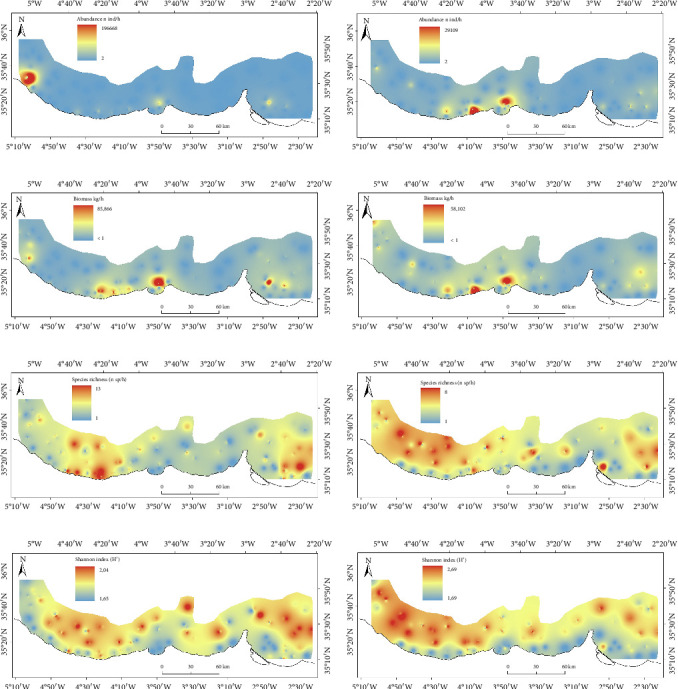
Crustaceans' cummunity distribution of abundance ((a) winter and (b) summer), biomass ((c) winter and (d) summer), species richness ((e) winter and (f) summer), and Shannon index ((g) winter and (h) summer), respectively (refer to Figure 1 for further details on the study area).

Abundance distribution patterns showed little differences between seasons (Figures 6(a) and 6(b)). A notable hotspot of abundance was observed in the central part of the study area, particularly near the coast during summer, while in winter, the abundance hotspot shifted to the western section of the continental shelf. Biomass distribution patterns mirrored those of abundance, with similar trends observed in both winter and summer (Figures 6(c) and 6(d)). Species richness distributions exhibited seasonal variations: higher biodiversity values were found closer to the upper slope in summer, whereas in winter, higher values were concentrated along the shoreline of the western part of the study area. Additionally, elevated concentrations of species richness were also noted in the eastern part of the region (Figures 6(e) and 6(f)). The distributions estimated using the Shannon index for the crustacean community exhibited patterns akin to those of species richness (Figures 6(g) and 6(h)). Notably, higher Shannon index values were observed as hotspots on the continental slope during the summer.

### 3.5. Bentho-Demersal Community–Environmental Relationships

The confrontation of different calculated indices and environmental variables highlights a highly significant relationship between the matrices (using Relate routine: Rho lower than 1; Table 4). The distribution of the different communities was influenced by combined spatial environmental parameters. The importance of these parameters in describing the distribution of separate community in the Moroccan coast is confirmed through the “Distance-based linear modeling” analysis (distLM) where each variable is examined individually.

**Figure 7:**
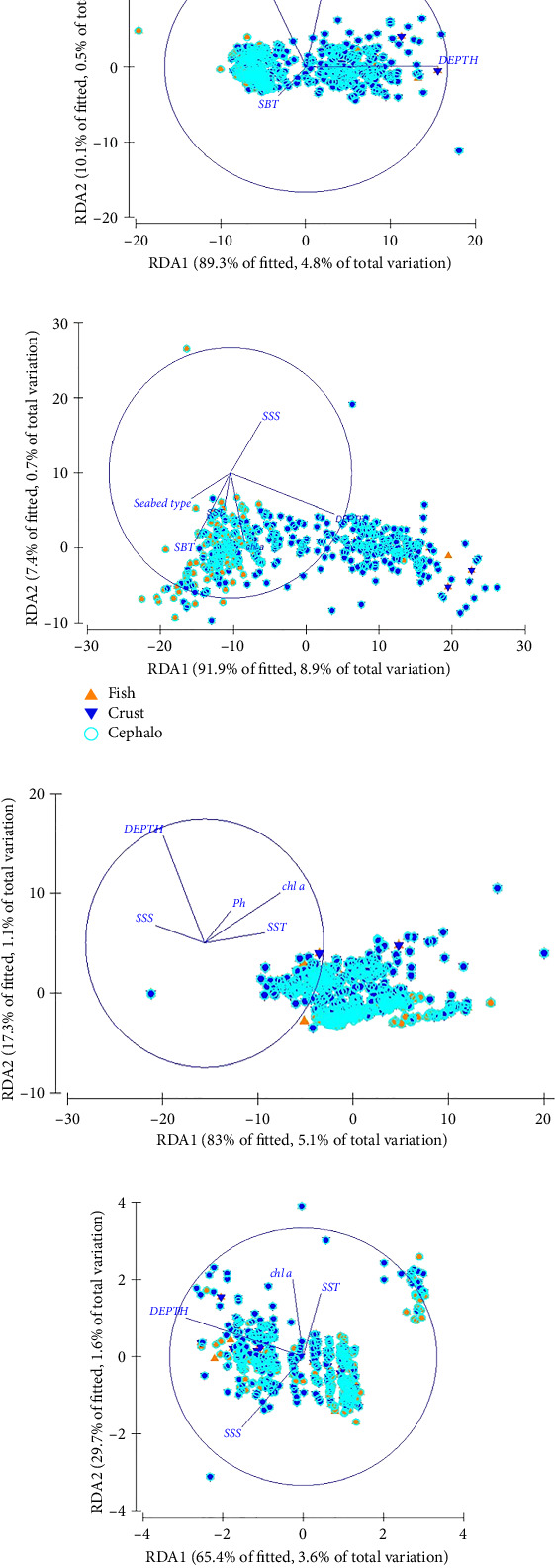
Distance-based redundancy analysis of log(*x*+1) transformed (a) abundance, (b) biomass, (c) richness index, and (d) biodiversity index data and standardized environmental data.

The primary two ordination axes (RDA1 and RDA2) accounted for the majority of the explained variation, with the proportion of Eigen value explaining this variation ranging from 65.4% to 91.9% (Figure 7). Among the investigated environmental variables, depth, temperature (SST and SBT), and salinity (SSS) for abundance and biomass, in addition to fluorescence (Chl a) for biodiversity indexes, exhibited a significant effect on the community composition, selected as the most suitable model (with the lowest AIC and BIC values), of the overall variation in the bentho-demersal community within the Moroccan Mediterranean (Table 3).

Depth emerged as the most influential factor in explaining the variation in the cephalopod community metrics, accounting for 39.7% of the variation in biomass, 25% in abundance, 59% in the richness index, and 13% in the Shannon index (DistLM, Table 4). For the crustacean community, distance-based linear modeling identified depth and SBT as the primary factors influencing community distribution. In contrast, SST, seabed nature, Chl a, DO, and pH had less significant effects (Table 4). Depth explained the largest proportion of variance in abundance (9.7%) and the Shannon index (17.1%), while SST accounted for 5.5% of the variance in biomass distribution and 9.88% in the richness index.

This analysis suggests that the differences observed in bentho-demersal community, from shallower to deeper waters, are primarily driven by increases in depth and salinity, alongside decreases in temperature, which delineate the distinct community structures.

## 4. Discussion

### 4.1. Abundance Fluctuation of Dominant Species

The cartographic representations of the results reveal that species are distributed along the Moroccan Mediterranean coast, yet their distribution varies significantly across the region. These maps reveal distinct spatial patterns in the distribution of bentho-demersal species, showcasing variability in their total abundance, biomass, and community diversity.

Significant concentrations are observed on either side of the Cape of Three Forks, especially in the Bay of Betoya and the central region. This area is renowned for its upwelling, a phenomenon caused by the interaction between Atlantic and Mediterranean water masses. This mixing boosts nutrient levels, leading to highly productive food webs that outpace those in other Mediterranean regions. The increased productivity not only sustains a wide variety of species but also establishes crucial spawning grounds for commercially important species. Additionally, these areas are close to river Kert and Ghis discharges, which have been demonstrated to be linked to high marine productivity [44]. These findings highlight the Moroccan Mediterranean coastline as a critical area of nursery and recruitment spot of many commercially valuable species.

When estimating the abundance and biomass within the total bentho-demersal community and for each specific category, the results showed remarkably similar spatial patterns. These findings provide compelling evidence of a strong spatial correlation. In addition, weaker but identifiable seasonal changes in the overall communities' structure were detected, mainly because of surges in a few species that either appeared in high numbers (due to spawning processes) or in significant biomass (due to access to abundant food resources) on the continental shelf. The spatial estimates of abundance and biomass for the bentho-demersal community revealed a general migration pattern from the upper slope towards the continental shelf. These seasonal movements within the study area are likely influenced by factors such as spawning, recruitment, nursing, or feeding events among deep-sea species. An increase in fish abundance during winter can be attributed to spawning activities, as many species migrate toward coastal zones to reproduce, leading to population spikes. These migrations are often driven by the search for better habitat conditions, ensuring food availability and higher survival rates for larvae [45]. In contrast, cephalopods showed a marked increase in abundance during the summer, primarily due to their reproductive cycle. This group tends to spawn in shallow waters during late winter, ensuring that their offspring hatch in warmer conditions, as noted in species like *Sepia officinalis* and *Octopus vulgaris* [46].

Regarding biomass for the three groups, higher values were observed during the winter season. This can be attributed to deep-sea species typically displaying an increased average weight due to their access to more abundant food resources and opportunities for self-repair and growth. Additionally, it is worth noting that areas where crustaceans were found, the presence of cephalopods was low. This observed pattern may be attributed to a reduction in predation pressure.

Similarly to abundance and biomass indices, the graphical representations of the evolution of the average number of species recorded per trawling stations during the study period shows that, in general, species richness decreases as depth increases (except for the crustacean's community: more details in spatial pattern of bentho-demersal community). This distribution is related to bathymetry, which demonstrated their spatial correlation. For instance, there is low species richness observed in the central area, similar to that seen offshore. Analysis of the collected data indicates that in this region, the seabed rapidly descends to a depth of 80 m within a span of less than 3 nautical miles from the shoreline [47]. However, higher values have been recorded for the crustacean community in upper slope (deep waters; contrary to that observed for fishes and cephalopods), indicating that certain groups of species do not follow this trend, and that sometimes significant depths (above 200 m) exhibit substantial richness, as is the case of offshore between M'diq and the Bay of Al-Hoceima. A previous study estimated diversity indices for the main resources exploited in the region and determined their spatial distribution. According to data collected from fisheries-independent scientific bottom trawl in spring season between 2006 and 2007 on the Moroccan Mediterranean [47], recorded between 1 and 33 species across the different trawling stations examined, with an average value of 20 species per station, indicating a relatively average specific richness. This paper recorded a greater species diversity, with 85 species identified in the winter compared to 62 species during summer. The rise in species richness can be partially explained by the wider geographic range and closer proximity of the sampling stations, as the study utilized 4 years of data. This extended temporal and spatial sampling allowed for the capture of a more diverse array of fish species.

### 4.2. Spatial Pattern of Bentho-Demersal Community

This study provides valuable insights for developing effective management and conservation strategies aimed at preserving the most diverse and ecologically significant communities.

While seasonal variations had minimal impact on the structure of these communities, clear spatial patterns were observed regardless of seasonal shifts. Depth emerged as a key factor driving the spatial structuring of communities, as demonstrated by dbRDA, which explained 10%–59% of the variation in ecological parameters for the three separate communities (Tables 3 and 4).

In our study, depth emerged as the most influential factor affecting abundance, biomass, and diversity, particularly within cephalopod and crustacean communities. Understanding the bathymetric distribution of commercially important species is essential for informed fisheries management. In Morocco, current management zones are defined by distance from shore while considering depth. Coastal and large-scale commercial fisheries are permanently prohibited from fishing within 1.5 miles of the coastline between Sebta and Al Hoceima, 2 miles between Al Hoceima and Cape Three Forks, and 3 miles from Cape Three Forks to Saidia. Small pelagic fisheries are restricted from fishing within 1 mile of the shore (Order No. 4202-14).

Although depth is often viewed as the primary factor driving changes in marine species distribution within shelf and slope ecosystems, its impact on species structure may also be tied to other environmental variables [48, 49]. Therefore, the spatial patterns observed in our study may result from a complex interplay of these factors. Previous studies have identified temperature and SSS as key environmental drivers that shape the spatial and temporal distribution of deep-sea communities. These factors play a critical role in species development, influencing metabolic rates and affecting the survival of eggs and juveniles through osmotic regulation. Significant shifts in these conditions can gradually alter the structure of marine communities over time [16, 21, 50]. In our case, we observed a positive correlation between temperature and both abundance and biodiversity across all three groups. Warmer temperatures appeared to promote greater species diversity, which can be attributed to the increased metabolic demand for food, leading to heightened competition and more dynamic predator–prey interactions [51, 52]. Temperature is a critical environmental factor for fish, as it regulates key physiological processes such as metabolism and growth [53, 54]. In our study, SSS was also significantly correlated with the fish community, likely due to its co-linearity with temperature (Figure 2).

Regarding fluorescence, earlier research [55] has demonstrated that elevated levels of primary production are closely linked to increased biodiversity, a pattern that aligns with our results. Notably, we found this positive relationship to be especially strong within the fish community, suggesting that increased primary production fosters higher biodiversity in this group.

Our dbRDA further confirmed that depth emerged as the principal environmental factor, while temperature, salinity, and Chl a contributed to a lesser extent for specific communities in shaping the overall structure in Morocco's coastal waters.

## 5. Management Implications

This study employed a quantitative approach to analyze the environmental impact on the spatio-temporal dynamics of species abundance and biodiversity within bentho-demersal communities (target and nontarget species). We examined a comprehensive dataset from 480 stations collected over 4 years to demonstrate the key characteristics of the studied system. This is especially important for the SW Mediterranean Sea, which is a hotspot for marine biodiversity, but unfortunately, is highly susceptible to extensive fishing, heavy maritime traffic, and a booming tourism industry. These factors are well documented to cause changes in communities, as well as to affect commercial properties. This issue is not unique to the Alboran Sea but is also observed in various other regions [56–60]. There is a pressing need to enhance our understanding of the composition of marine communities and their relationship with environmental factors. This knowledge is crucial for evaluating the effects of human activities, such as fishing, the duration of these effects, and the subsequent recovery of the affected communities.

Current research indicates that all examined communities are affected by fishing and other human activities [47], although the full extent of these impacts on community structure remains unclear. Previous studies have shown that intense fishing pressure can directly or indirectly alter the structure of bentho-demersal fish assemblages [58, 61, 62].

Our findings largely confirm that bentho-demersal communities along the Moroccan coast respond to environmental gradients from offshore to inshore, with minimal seasonal variation. This suggests that spatial management measures are likely to be more effective than temporal restrictions and should be prioritized in sustainable fisheries strategies.

Such measures can help prevent overfishing and mitigate its effects on marine resources, ecosystems, and communities, which aligns with the principles of an Ecosystem Approach to Fisheries Management [63]. This study provides valuable information for managers, scientists, and fishermen, facilitating a clearer view of how the dynamics of harvested communities respond to fishing activities and environmental changes. This knowledge is crucial for making informed decisions on resource management and conservation strategies.

## 6. Conclusion

Community analyses offer crucial insights for fisheries management, like bottom *trawling* in Moroccan Mediterranean. They help in: (i) delineating spatial and temporal variations in bentho-demersal communities, (ii) identifying subcomponents of fisheries experiencing similar conditions concurrently, such as fishing pressure and environmental changes, and (iii) creating management strategies to separate various fisheries or fishing methods based on the spatial communities' patterns.

In this study, clear patterns emerged, demonstrating that spatial structuring is a significant factor affecting the communities across all examined area, with depth identified as a potential driving force. To manage fishing efforts effectively, it is crucial to consider this spatial structuring, which includes the distribution and arrangement of marine species when establishing fishing boundaries and regulations. Management strategies should incorporate depth as a key consideration when revising existing fishing zones, rather than relying solely on distance from shore. For instance, in Morocco, current fishing zones based on depth align with the assemblage patterns observed in this study.

To manage the cumulative impact of various fishing practices and gear types, it is essential to design management zones that reflect the spatial distribution of marine resources. While distance from shore might be easier to enforce, it should correspond to relevant depths and assemblages to ensure ecological accuracy. It is important to note that distance from shore does not always accurately reflect ecological conditions; specific distances may correspond with particular depths or marine communities that are more meaningful ecologically.

In conclusion, conducting thorough regional assessments of communities' structures using existing scientific trawl survey data can provide valuable insights. Future efforts should focus on analyzing temporal changes in local and regional assemblages to assess their stability and understand the impacts of human activities, especially fishing.

## Figures and Tables

**Table 1:** Overview of the variables included as potential fixed effects examined for their influence on the ecological metrics of the bentho-demersal communities.

Variable	Description	Units	Source
SST	The temperature of the water's surface	°C	Copernicus
SBT	The temperature of the bottom	°C	Copernicus
SSS	Salt concentration of the surface waters	PSS	Copernicus
PH	The acidity or alkalinity of the water	—	Copernicus
DO	Oxygen available in the water	mg/L	Copernicus
Chl a	Phytoplankton concentration in the sampling location	μg Chl L^−1^	Copernicus
Seabed nature	The type of substrate on the seabed	Mud, sand, and rock	Survey
Season	When the sample was collected	Winter, summer	Survey
Depth	Depth at which samples were collected	Meters	Survey

**Table 2:** Average (avr), median (med), SD, minimum (min) and maximum (max) values of observed abundance (n h^−1^), observed biomass (kg h^−1^), observed richness index (n haul^−1^), and observed Shannon index of the total demersal community and of fish, cephalopod, and crustacean assemblages in both seasons (winter and summer).

		Winter					Summer					P (K-W)	P (W)
		Avr	Med	SD	Min	Max	Avr	Med	SD	Min	Max		
Total	Abundance	5894.81	2008.5	15687.93	7	207,156	3155.84	1588	5659.23	16.1	47,522	**< 0.05**	**< 0.05**
	Biomass	92.907	57.199	117.681	0.10	753	64.420	44.093	68.975	0.640	566.68	**< 0.05**	**< 0.05**
	Richness	33.72	30	15.77	2	85.71	31.23	28	13.36	6	62	0.106	**< 0.05**
	Shannon	1.53	1.61	0.57	0.01	2.58	1.55	1.62	0.58	0.02	2.76	0.714	0.862

Fish	Abundance	3827.43	1386	7033.55	2	64,798	2353.05	1178	3878.18	10.25	33,334	0.104	**< 0.05**
	Biomass	76.914	44.161	113.751	< 1	753	55.164	34.406	65.223	< 1	528.38	**< 0.05**	**< 0.05**
	Richness	24.67	24	12.28	1	58	24.24	22	11.33	3	54	0.77	0.246
	Shannon	1.34	1.47	0.53	0	2.34	1.34	1.50	0.59	0.02	2.48	0.993	0.128

Cephalopod	Abundance	148.96	56	255.45	1	2000	311.35	99	528.01	1	3822	**< 0.05**	**< 0.05**
	Biomass	14,804	5384	19,231	< 1	104.56	8921	5338	9616	< 1	57,460	0.759	**< 0.05**
	Richness	6.74	6	4.57	1	22.86	6.03	5	3.89	1	16	0.238	**< 0.05**
	Shannon	0.80	0.84	0.50	0	1.84	0.57	0.51	0.43	0	1.75	**< 0.05**	0.062

Crustacean	Abundance	2306.47	95.4	16,132.43	2	202,776	854.28	53	3294.97	1	29,260	0.182	**< 0.05**
	Biomass	5463	1379	13,074	< 1	134.74	3508	1380	7271	< 1	58.4	0.582	**< 0.05**
	Richness	4.58	4	2.42	1	14	4.01	4	1.84	1	9	0.069	**< 0.05**
	Shannon	0.61	0.63	0.49	0	1.73	0.64	0.62	0.53	0	1.82	0.661	0.086

*Note:p* values in bold are statistically significant.

**Table 3:** Summary of best selected model using Akaike information criterion (AIC) and Bayesian information criterion (BIC) for response variables based on the dbRDA.

		Model	AICC	BIC
Total	Abundance	**DEPTH**⁣^∗^ + **Chl a** + **SST** + SBT	8469	8483.4
	Biomass	**DEPTH**⁣^∗^ + **SSS + SBT + seabed + Chl a** + **SST**	9125	9141.4
	Richness	**Chl a**⁣^∗^ + **SST**⁣^∗^ + **DEPTH**⁣^∗^ + **SSS + PH**	7583.1	7607.4
	Shannon I	**DEPTH**⁣^∗^ + **SSS**⁣^∗^ + **Chl a** + **SST**	4388.3	4411.8

Fish	Abundance	**SBT**⁣^∗^ + **SSS**⁣^∗^ + **seabed**⁣^∗^ + **Chl a** + DO	3132.4	3152.1
	Biomass	**SSS**⁣^∗^ + **SBT** + seabed	3071.8	3082.9
	Richness	**SBT + seabed + SSS + DO + PH**	2370.4	2473.3
	Shannon I	**Chl a**⁣^∗^ + **seabed + SSS**	2611.4	2621.8

Crust	Abundance	**DEPTH**⁣^∗^ + **SBT**⁣^∗^ + **SST + PH + seabed + Chl a** + **SSS**	2479.1	2501.2
	Biomass	**SBT**⁣^∗^ + **DEPTH**⁣^∗^ + Chl a	2374.9	2386.4
	Richness	**SBT**⁣^∗^ + **SST**⁣^∗^ + DEPTH	1696.1	1707.8
	Shannon I	**DEPTH**⁣^∗^ + **DO**⁣^∗^ + **SST + SBT**	1488.2	1500.9

Cephalo	Abundance	**DEPTH**⁣^∗^ + **SBT**⁣^∗^ + **SST + PH + seabed + Chl a** + **SSS**	2479.1	2501.2
	Biomass	**SBT**⁣^∗^ + **DEPTH**⁣^∗^ + Chl a	2374.9	2386.4
	Richness	**SBT**⁣^∗^ + **SST**⁣^∗^ + DEPTH	1696.1	1707.8
	Shannon I	**DEPTH**⁣^∗^ + **DO**⁣^∗^ + **SST + SBT**	1488.2	1500.9

*Note:* The significant environment variables were presented in bold.

⁣^∗^Selected variable by BIC.

**Table 4:** Results of distance-based multivariate linear model (DistLM) for bentho-demersal species biomass, abundance, richness index, and Shannon index showing the percentage variation explained by best-selected environmental variables.

**Variables**	**% Explained variation (fitted model)**		**% Explained variation (Total)**		**Variables**	**% Explained variation (fitted model)**		**% Explained variation (Total)**	
**Biomass**					**Richness index**				

*Fish* *Rho: 0.002*	*Indiv*	*Cum.*	*Indiv*	*Cum.*	*Fish* *Rho: 0.076*	*Indiv*	*Cum.*	*Indiv*	*Cum.*
SSS	96.4	96.4	4.77	4.77	SBT	99.39	99.39	23.72	23.72
SBT	2.95	99.35	0.15	4.92	Seabed	0.61	100	0.15	23.87
Seabed	0.65	100	0.03	4.95	SSS	0	100	0	23.87

*Cephalopod* *Rho: 0.15*	*Indiv*	*Cum.*	*Indiv*	*Cum.*	*Cephalopod* *Rho: 0.232*	*Indiv*	*Cum.*	*Indiv*	*Cum.*
Depth	96.6	96.6	39.7	39.7	Depth	99.67	99.67	58.9	58.9
SST	3.32	99.91	1.36	41.06	Chl a	0.33	100	0.2	59.1
Chl a	0.07	99.98	0.03	41.09					
Seabed	0.02	100	0.01	41.1					

*Crustacean* *Rho: 0.067*	*Indiv*	*Cum.*	*Indiv*	*Cum.*	*Crustacean* *Rho: 0.11*	*Indiv*	*Cum.*	*Indiv*	*Cum.*
SBT	74.9	74.9	5.49	5.49	SBT	99.25	99.25	8.88	9.88
DEPTH	25.02	99.93	1.83	7.32	SST	0 .74	99.99	0.07	8.95
Chl a	0.07	100	0.01	7.33	DEPTH	0 .01	100	0	8.95

**Abundance**					**Shannon index**				

*Fish* *Rho: 0.063*	*Indiv*	*Cum.*	*Indiv*	*Cum.*	*Fish* *Rho: 0.039*	*Indiv*	*Cum.*	*Indiv*	*Cum.*
SBT	96.09	96.09	14	14	Chl a	94.47	94.47	3.04	3.04
SSS	3.66	99.75	0.53	14.54	Seabed	5.43	99.9	0.17	3.22
Seabed	0.21	99.96	0.03	14.75	SSS	0.1	100	0	3.22
Chl a	0.04	100	0.01	14.57					

*Cephalopod* *Rho: 0.079*	*Indiv*	*Cum.*	*Indiv*	*Cum.*	*Cephalopod* *Rho: 0.034*	*Indiv*	*Cum.*	*Indiv*	*Cum.*
Depth	99.37	99.37	25.04	25.04	Depth	98.91	98.91	12.99	12.99
Chl a	0.48	99.85	0.12	25.16	Chl a	1.05	99.96	0.14	13.13
SST	0.15	100	0.04	25.2	SST	0.04	100	0.01	13.14

*Crustacean* *Rho: 0.07*	*Indiv*	*Cum.*	*Indiv*	*Cum.*	*Crustacean* *Rho: 0.137*	*Indiv*	*Cum.*	*Indiv*	*Cum.*
Depth	66.32	66.32	9.64	9.64	Depth	99.41	99.41	17.1	17.1
SBT	32.78	99.1	4.76	14.4	DO	0.34	99.74	0.06	17.15
SST	0.68	99.78	0.1	14.5	SST	0.25	100	0.04	17.2
Ph	0.14	99.93	0.02	14.52	SBT	0	100	0	17.2
Seabed	0.06	99.98	0.01	14.53					
Chl a	0.02	100	0	14.53					

## Data Availability

The datasets from trawl scientific used and analyzed to support the findings of this study have not been made available due to confidentiality and cannot be shared due to institutional and legal restrictions.
